# Biodegradation of Diesel by Free and Immobilized *Chromobacterium violaceum*

**DOI:** 10.1021/acsomega.5c07846

**Published:** 2025-11-12

**Authors:** Jhonson Sebastian Arenas Soler, Nathaly Rivera, Francy Janeth Méndez Casallas, Boris Galvis

**Affiliations:** † Programa de Ingeniería Ambiental Y Sanitaria, 27990 Universidad de La Salle, Bogotá 111711, Colombia; ‡ Facultad de Ciencias de la Salud, Universidad Colegio Mayor de Cundinamarca, Bogotá 110911, Colombia; § Sanitary and Environmental Engineering Program, Universidad del Valle, Cali 760042, Colombia

## Abstract

Pollution from oil and its derivatives has significantly affected terrestrial and marine ecosystems around the world. The use of microorganisms with the ability to biodegrade these contaminants has been proven as an alternative to remediate contaminated sites. This research estimated the biodegradation efficiency of Total Petroleum Hydrocarbons (TPHs) and phenols present in a commercial sample of diesel by *Chromobacterium violaceum* ATCC 12472 at a laboratory scale. We quantified the decrease in the concentration of phenols and TPHs by *C. violaceum* by free and immobilized cells in calcium alginate beads, as well as by *Pseudomonas aeruginosa* ATCC 9027 (positive control). The biodegradation of TPHs by *C. violaceum* during 10 days of treatment was 10% in free cells and 19% in immobilized cells, while for *P. aeruginosa*, it was 16 and 25%, respectively. In relation to phenols, biodegradation by free cells of *C. violaceum* was 83% and 86% by immobilized cells, while *P. aeruginosa* was 83 and 85%, respectively. These results indicate that cell immobilization enhanced the biodegradation of TPHs but not of phenols. Therefore, *C. violaceum* is a good alternative for the treatment of process water with hydrocarbons almost as efficient as *P. aeruginosa*. Additionally, the immobilization of cells in calcium alginate beads represents an alternative to obtain greater efficiencies in the removal of TPHs.

## Graphical abstract

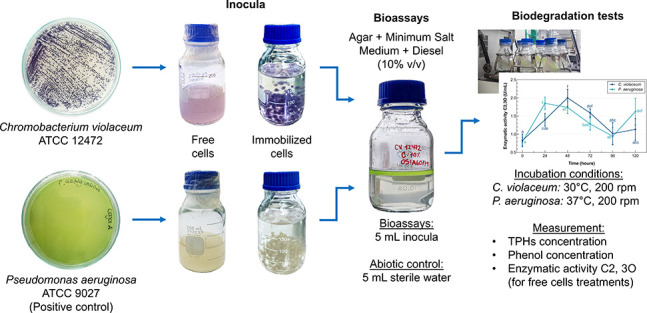

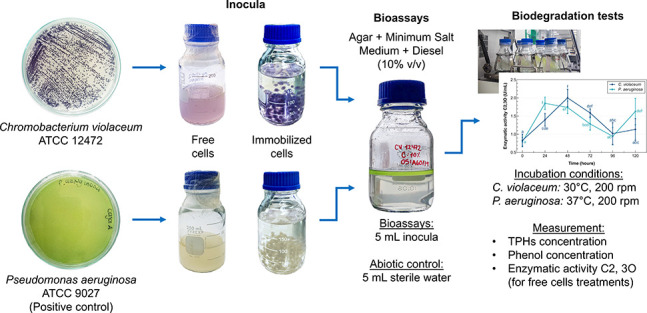

## 1 Introduction

Oily sludges from the hydrocarbon industry are a complex mixture of cycloalkanes, benzene, polycyclic aromatic hydrocarbons, and an aqueous phase with organic matter, limited nutrients, and some dissolved metals. They also contain a solid phase composed of inorganic solids, mixed oxides, and amorphous carbon.^1–4^

These sludges pose significant challenges: hydrocarbons are difficult to recover, the contaminated water is hard to treat, and disposing of the remaining residue is cumbersome.^5–8^ Additionally, sludge storage consumes valuable space at many industrial sites^3^ and improper management can lead to severe environmental consequences.^9,10^

Bioremediation is one of several methods for cleaning up contaminated oily sludges.^4,11^ To ensure compliance with environmental regulations, the successful application of this and other treatment technologies must achieve a balance between cost and effectiveness.^3,6^ For bioremediation technologies this balance depends mainly on the metabolic capacity of microorganisms to degrade hydrocarbons.^9,12^

Many research efforts have identified bacteria with favorable characteristics for bioremediation of hydrocarbons. *Pseudomonas*, *Bacillus*, and *Acinetobacter* species can efficiently biodegrade hydrocarbons, including crude oil and polycyclic aromatic hydrocarbons, with efficiencies ranging from 16% to 99%, depending on conditions like temperature, pH, and the presence of biosurfactants and nutrients.^13–20^

Diesel has been often used as substrate for these experiments because it contains aliphatic and aromatic hydrocarbons, with a high concentration of monoaromatic hydrocarbons and polycyclic aromatic hydrocarbons.^21,22^ In addition, the composition of diesel includes stabilizing substances, which are added to prevent oxidation of the fuel and prevent the formation of particles that can be deposited in the injection system of the engines. Among the most used additives are phenylenediamine, and phenols such as 2,6-di*tert*-butyl-4-methylphenol, generally in the range of 10 to 80 ppm.^23,24^

In diesel degradation, *n*-alkanes are the most easily degraded compounds within the aliphatic hydrocarbon group.^25^ The specific enzymes responsible for their breakdown depend on the molecular weight of the *n*-alkane. For low-molecular-weight gaseous alkanes (C_1_–C_4_), methane monooxygenase is the primary enzyme involved, whereas for medium-chain alkanes (C_5_–C_16_), degradation is mainly carried out by alkane monooxygenase.^26^

Phenols are one of the main organic pollutants, as well as one of the most dangerous, that are discharged into wastewater from the refining of crude oil.^27,28^ They are obtained during physical separation processes by fractional distillation of petroleum and are known to be highly toxic even at low concentrations. Their presence in natural water bodies can lead to the formation of other, even more toxic compounds.^29–32^

The microbial degradation of aromatic compounds such as phenol, can occur through the *ortho-* or *meta-route*. The role played by the dioxygenase enzymes intradiol and estradiol for the breakdown of the aromatic ring is key in this process.^33–35^ Therefore, the measurement of the activity of this enzyme is essential to understand the degradation process of phenols and other aromatic compounds. Catechol 2,3-dioxygenase (C2,3O) an estradiol dioxygenase has been widely studied for its action on meta-cleavage of the phenolic ring.^33^ This enzyme has been found in numerous strains of Gram-negative bacteria such as *Pseudomonas*, *Acinetobacter*, *Ralstonia*, *Burkholderia*, *Stenotrophomonas*, and *Sphingomonas* can express C2,3O to facilitate the breakdown of phenols.^36–40^

The biodegradation of diesel is influenced by both the concentration and specific composition of its hydrocarbons. Certain compounds, when present at particular concentrations, can inhibit bacterial growth, reduce biodegradation efficiency, or even cause bacterial death.^22,41,42^ To overcome these challenges and enhance microbial degradation capacity, several strategies have been developed. These include progressive acclimatization of microbes to contaminants, genetic modification, and cell immobilization techniques.^43–45^ The cell immobilization approach involves using various media or support materials (biofilms) that protect microorganisms not only from the toxic compounds being treated, but also from environmental threats such as bacteriophages, protozoa, and toxins.^46,47^ Among the available immobilization polymers, alginate is one of the most used due to several advantages: (i) it allows for a simple immobilization process, (ii) it can be easily degraded through chemical or enzymatic hydrolysis, or by radiation, and (iii) it has low toxicity.^48^

The number of bacterial strains reported in the literature capable of growing at high concentrations of diesel is quite limited, because it is generally difficult for them to tolerate the inherent toxicity of the medium and to exhibit degrading abilities for all components of this fuel.^49^ Several of the bacterial genera that have been found to be able to degrade diesel have metabolic versatility, being able to use both aliphatic and aromatic hydrocarbons. Hydrocarbonclastic bacterial populations that persist in contaminated environments show good diesel bioremediation potential.^50^ Among the group of bacteria that have been reported in places contaminated with hydrocarbons is *Chromobacterium violaceum*.^51^ The genome sequence of *C. violaceum* ATCC 12472 generated in 2003 shows that this bacterium has a versatile metabolism capable of exploring a wide range of energy sources using oxidases and reductases, which allows it to survive under various environmental conditions.^52^

Previous studies demonstrated that *C. violaceum* can grow in high diesel concentrations (10% v/v), highlighting its potential for diesel degradation.^53^ Building on these findings, the present study aimed to evaluate the biodegradation efficiency TPHs and phenols in a commercial diesel sample using both free and immobilized cells of *C. violaceum* ATCC 12472 at the laboratory scale.

## 2 Materials and Methods

### 2.1 Reactivation of Strains and Preparation of Inoculum

*C. violaceum* ATCC 12472 and *P. aeruginosa* ATCC 9027 were reactivated in agar–diesel (prepared with Minimum Salt Medium (MSM) + agar–agar 1.5% m/v + diesel 10% v/v) from vials obtained in a previous study,^53^ where the strains had been conditioned in diesel media. To ensure the absence of another microorganism in the cultures, the growth medium was sterilized in an autoclave. Table 1 indicates the composition of MSM, which was established previously.^54^

**Table 1:** Composition of the Minimum Salt Medium (MSM)

Species	Concentration (mg/L)
CaCl_3_	1
NaHCO_3_	125
K_2_SO_4_	70
NaH_4_NO_3_	70
KH_2_PO_4_	100
MgSO_4_–7H_2_O	10
MnCl_2_–H_2_O	7
ZnSO_4_	1.5

Cultures of *C. violaceum* ATCC 12472 and *P. aeruginosa* ATCC 9027 in agar–diesel were incubated for 24 h at 30 and 37 °C, respectively. After 72 h of incubation, each strain was transferred to a Schott bottle with 99 mL of sterile MSM and 1 mL of diesel. The inoculums were incubated again for 72 h in the Thermo Scientific MAXQ 4450 orbital shaker at 200 rpm, maintaining the same temperature for each strain. Subsequently, the inoculums were spectrophotometrically adjusted to 600 nm to obtain a bacterial concentration of 5 × 10^8^ CFU/mL using McFarland standards.

### 2.2 Immobilization of Strains in Calcium Alginate Beads

The adapted strains of *C. violaceum* ATCC 12472 and *P. aeruginosa* ATCC 9027 were immobilized by entrapment in a calcium alginate matrix, taking as a reference the methodology carried out by Urbina and Dussan-G^55^ with modifications. In 50 mL of each inoculum, 1 g of sodium alginate was dissolved. For each strain, 5 mL of the mixture was taken using a sterile 10 mL commercial syringe and slowly dripped from the syringe orifice (0.6–0.8 mm in diameter; 21G or 23G), into a cold sterile 2% calcium chloride solution. As the droplets fell, the calcium chloride solution stirred, allowing the beads to form. Abiotic control was performed in the same way, taking 50 mL of sterile water instead of inoculum. The beads were washed with 50 mL of sterile water to remove excess Ca^2+^ ions.

### 2.3 Biodegradation Tests of TPHs and Phenols by Free and Immobilized Cells of *C. violaceum* ATCC 12472 and *P. aeruginosa* ATCC 9027

Biodegradation assays were performed in 500 mL Schott bottles with 250 mL of sample, to allow oxygenation of the sample in a 1:1 ratio. The bioassays consisted of two treatments (one with free cells and the other with immobilized cells), each with its respective abiotic control and in triplicate. The composition of the samples consisted of 5 mL of bacteria for both treatments, 25 mL of diesel and the rest of sterile MSM. The concentration of diesel used was 10% v/v, corresponding to the maximum tolerance found in the previous study.^53^

Abiotic controls were prepared using sterile water (for the free cell treatment) and sterile beads (for the immobilized cell treatment) in place of the microbial inoculum. All treatments were continuously incubated in a Thermo Scientific MAXQ 4450 orbital shaker at 200 rpm,^54^ and at a temperature of 30 °C for *C. violaceum* treatments and 37 °C for *P. aeruginosa* treatments. The assay was conducted for a period of 10 days—corresponding to the time at which *C. violaceum* ATCC 12472 reaches its stationary growth phase. Biodegradation was assessed by directly measuring the reduction in phenol and total petroleum hydrocarbon (TPH) concentrations, and indirectly by monitoring the increase in enzymatic activity of C2,3O.

### 2.4 Measurement of the Concentration of TPHs and Phenols in Batch Cultures of *C. violaceum* ATCC 12472 and *P. aeruginosa* ATCC 9027

TPH concentrations were sampled at the beginning and end of assembly, while phenol concentrations were sampled daily until the highest degree of degradation was reached. Both were made based on the procedures established in Standard Methods for Examination of Water and Wastewater.^56^ The laboratory analysis was conducted by ANALQUIM LTDA (Bogota, Colombia), which is certified by the Colombian Institute of Hydrology, Meteorology and Environmental Studies (IDEAM). The analysis followed the standard Soxhlet extraction method, as described in Standard Methods 5520 D and F. Phenol concentrations were determined using the 4-aminoantipyrine colorimetric method (Standard Methods 5530 D), with specific modifications detailed below.

Pretreatment was initially applied to the sample to eliminate potential interferences with the analytical technique. One milliliter of the sample was placed in a microcentrifuge tube, and 100 μL of 10% H_3_PO_4_ was added. The mixture was centrifuged at 5000 rpm for 5 min using a SCILOGEX D2012 Plus microcentrifuge. The resulting supernatant was then diluted with distilled water to various concentrations, ensuring the final volume was always adjusted to 5 mL and remained within the method’s measurement range.

Because the samples contained fuel oil, a chloroform extraction was necessary. Approximately one-quarter of a ball of NaOH was added to each tube to raise the pH to 12. Then, 2.5 mL of chloroform was added to each sample, resulting in the formation of two distinct phases. The lower phase was discarded, and the upper aqueous phase was placed in a water bath at 62 °C for 5 min to evaporate any residual chloroform.

Next, 250 μL of 2 N NH_4_OH was added to each sample, followed by gentle mixing. This step was also performed for the blank, using 5 mL of distilled water instead of the sample. Afterward, 125 μL of 2% 4-aminoantipyrine was added to each tube, mixed again, and then 125 μL of 8% potassium ferricyanide [K_3_Fe­(CN)_6_] was added. The mixtures were vigorously stirred and allowed to stand for 15 min for color development.

Finally, absorbance readings were taken at 510 nm using a HACH DR-1900 spectrophotometer. Phenol concentrations were calculated by interpolating the absorbance values into the linear regression model of the calibration curve (see Supporting Information), with appropriate correction for the dilution factors applied.^56,57^ The same procedure was used to determine the total phenol concentration in the commercial diesel sample. To assess whether there were statistically significant differences between treatments, a one-way analysis of variance (ANOVA) was conducted using IBM SPSS Statistics software (version 25) (see Supporting Information).

### 2.5 Phenol Calibration Curve

The calibration curve for the determination of phenol concentration was made from five standard solutions. The standard solutions were prepared at 1, 2, 3, 4, and 5 ppm and each was processed according to the 4-aminoantipyrine method (Standard Methods 5530 D). Absorbances corresponding to each standard were measured on the UV–visible spectrophotometer at 510 nm.^56^ The data were analyzed using the linear regression model statistical software GraphPad Prism (version 8.0.1) (see Supporting Information).

### 2.6 Enzymatic Activity of C2,3O by Free Cells of *C. violaceum* ATCC 12472 and *P. aeruginosa* ATCC 9027

The activity of the C2,3O enzyme was assessed only in the treatment with free cells, as its measurement in immobilized cells posed significant challenges. Since C2,3O is an intracellular protein, analyzing its activity required releasing the cells from the immobilization matrix, which was not feasible under the experimental conditions. Enzyme activity was measured spectrophotometrically following the methodology described by Olukunle et al.,^58^ with the modifications detailed below.

To extract proteins, 1 mL of the sample was centrifuged at 14,000 rpm for 5 min using a SCILOGEX D2012 Plus microcentrifuge. The supernatant was discarded, and the resulting pellet was resuspended in 1 mL of Trisaminomethane-sucrose buffer (30 mM Trisaminomethane-HCl, pH 8.0, with 20% sucrose). Next, 10 μL of 0.5 M EDTA (pH 8.0) and 100 μL of lysozyme solution (2.5 mg/mL in Trisaminomethane-sucrose buffer) were added. The mixture was incubated on ice for 30 min and then centrifuged at 12,000 rpm for 5 min. The resulting pellet was resuspended in 200 μL of lysis buffer (30 mM Trisaminomethane-HCl, pH 8.0, and 5 mM MgCl_2_).

To determine the activity of C2,3O, the 200 μL of the resulting protein fraction was taken and served in a 10 mL test tube. 400 μL of catechol was added at 0.1 mM and completed at 4 mL of reaction with pH 7.6 phosphate buffer. Targeting was done by adding an additional 200 μL of catechol to 0.1 mM in substitution of protein. The absorbance generated with the HACH DR-1900 spectrophotometer was measured and incubated at 30 °C for 30 min. After this time, the absorbance was measured again.

To determine C2,3O activity, 200 μL of the extracted protein fraction was transferred to a 10 mL test tube. Then, 400 μL of 0.1 mM catechol was added, and the reaction volume was brought to 4 mL using phosphate buffer at pH 7.6. Targeting was done by adding an additional 200 μL of catechol to 0.1 mM in substitution of protein under the same conditions. Absorbance was measured using a HACH DR-1900 spectrophotometer, after which the mixture was incubated at 30 °C for 30 min. Absorbance was measured again at the end of the incubation period to evaluate enzyme activity.

The calculation of the enzymatic activity was made by the increase in absorbance at 375 nm caused by the accumulation of 2-hydroxymuconic semialdehyde, taking into account that 1 enzyme unit is the amount of protein produced by 1 μmol of 2-hydroxymuconic semialdehyde in 1 min and 1 μmol of this compound increases the absorbance at 375 nm in 14.7. To assess the existence of significant differences in the enzymatic activity of C2,3O between treatments, an ANOVA was performed using IBM SPSS Statistics software (version 25) (see Supporting Information).

### 2.7 Calculation of the Biodegradation of TPHs and Phenols by Free and Immobilized Cells of *C. violaceum* ATCC 12472 and *P. aeruginosa* ATCC 9027

The percentages of biodegradation of TPHs and phenols for each treatment were calculated based on the procedure described by Narvaez Florez et al.,^54^ as the difference between the biotic and abiotic degradation percentages (eqs 1 and 2). Additionally, the rate of phenol biodegradation between treatments was calculated.

1 $$\%\;\mathrm{Total}\;\mathrm{degradation}=100\%-\left(\frac{\mathrm{Final}\;\mathrm{Concentration}}{\mathrm{Initial}\;\mathrm{Concentration}}\times 100\%\right)$$

2 %Biodegradation=%BioticDegradation−%AbioticDegradation

## 3 Results and Discussion

### 3.1 Biodegradation of TPHs by *C. violaceum* ATCC 12472

Based on the previously evaluated growth kinetics of *C. violaceum* ATCC 12472 at its maximum tolerance to diesel (10% v/v),^53^ the TPH biodegradation test was conducted over a 10-day incubation period, corresponding to the point at which the culture reached its stationary phase and achieved maximum biodegradation efficiency. The exponential and stationary phases are critical for assessing the highest biodegradation rates, as the bacterium remains metabolically active during these stages.

According to Rolfe et al.,^59^ cell division occurs at a constant rate during exponential growth, so in batch culture systems the greatest increase in biomass is observed within this phase. In the exponential phase, the bacterium uses the TPHs present in diesel to synthesize the necessary macromolecules through replication, which involves DNA synthesis along with transcription and translation. Subsequently, during the stationary phase, other compounds continue to be metabolized because cells try to gain biomass in the shortest possible time to secure resources before their competitors.^60^

Figure 1 shows the differences between TPHs concentrations at the start and end of biodegradation assays. As observed there, treatment with immobilized cells of both bacteria presented a greater decrease in the concentration of TPHs than treatments with free cells. Additionally, *P. aeruginosa* ATCC 12472 presented a higher biodegradation rate than *C. violaceum* ATCC 12472. The total concentration of TPHs in the initial diesel sample is 725 ± 23 g/L.

**Figure 1:**
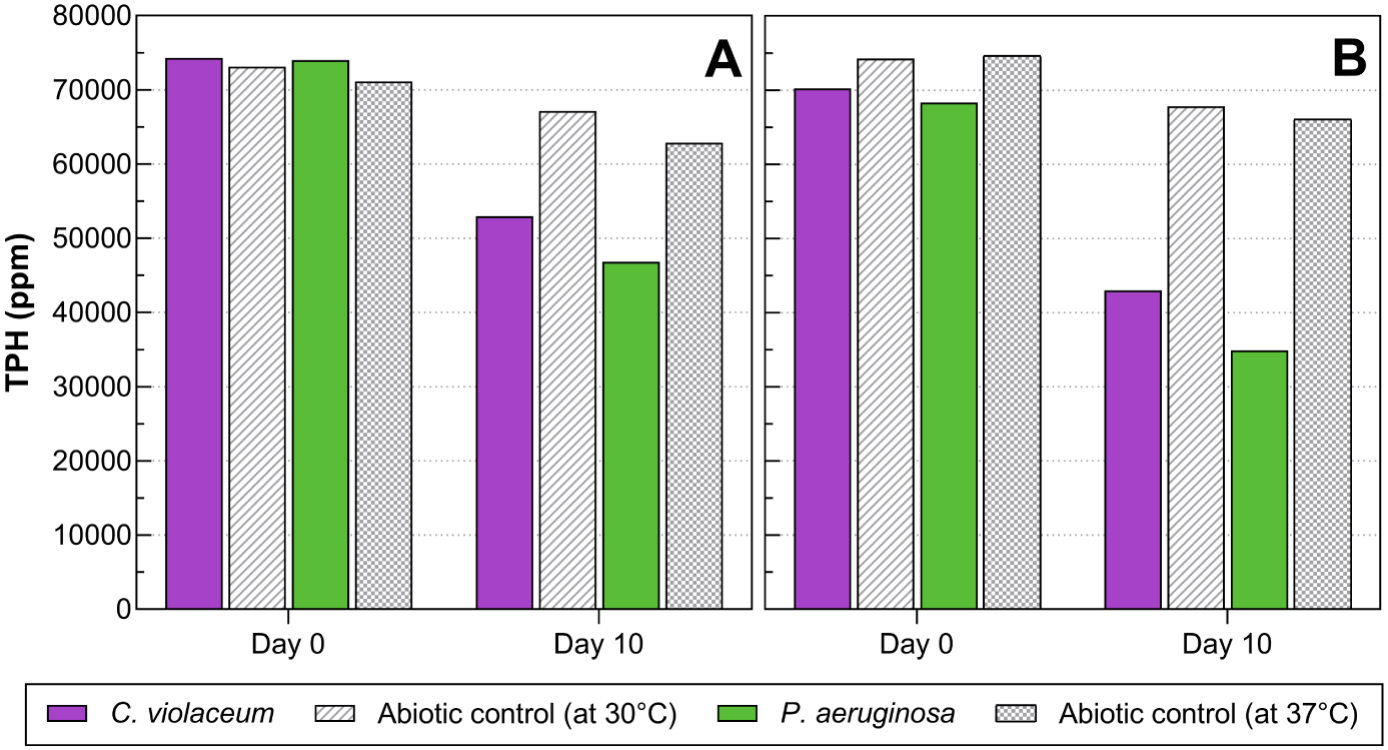
Degradation of TPHs by free (A) and immobilized (B) cells of *C. violaceum* ATCC 12472 and *P. aeruginosa* ATCC 9027 (positive control).

Treatment with immobilized cells of *P. aeruginosa* ATCC 9027 presented the highest percentage of TPHs degradation (49%), 25% of it attributed to bacterial metabolism and 24% of removal associated with abiotic factors (see Table 2). Considering the test conditions, the 24% decrease in abiotic control is a product of the reaction of TPHs with oxygen. On the other hand, cell immobilization of *C. violaceum* ATCC 12472 and *P. aeruginosa* ATCC 9027 increased the biodegradation rate of TPHs by 0.89 and 0.90%, respectively.

**Table 2:** Percentages of Biodegradation of TPHs by Free and Immobilized Cells of *C. violaceum* ATCC 12472 and *P. aeruginosa* ATCC 9027 (Positive Control) 10 Days after the Test

Treatment	Bacterium	Biotic degradation (%)	Abiotic degradation (%)	Biodegradation (%)
Free	*C. violaceum* ATCC 12472	29	19	10
	*P. aeruginosa* ATCC 9027	37	22	16
Immobilized	*C. violaceum* ATCC 12472	42	23	19
	*P. aeruginosa* ATCC 9027	49	24	25

The decrease in the concentration of TPHs after 10 days of incubation (Figure 1) and the progressive increase in biomass in previous studies^53^ is consistent with the results presented by Ichor et al.,^61^ in which bacteria isolated from Bodo Creek in Nigeria, belonging to the families *Alcaligenaceae*, *Enterobacteriaceae, Flavobactericeae, Planococcaceae, Pseudomonadaceae* and *Bacilliceae*, showed a negative correlation between bacterial growth and the decrease in the concentration of TPHs in free environment and aerobic conditions. Therefore, it is possible to point out that the degradation of TPHs in the diesel sample is directly related to bacterial metabolism.

Regarding treatments with immobilized cells, the results found are similar to those reported in the literature, where the encapsulation of cells in calcium alginate beads facilitates and increases the rate of biodegradation of hydrocarbons. This can be attributed to the fact that the immobilization process in this medium increases the density of bacterial cells and, consequently, there is a higher metabolic activity of microorganisms in bioreactors.^62^

Our study demonstrated enhanced hydrocarbon removal efficiency when *Pseudomonas* spp. was used in immobilized media (see Figure 1 and Table 2). The hydrocarbon biodegradation mechanisms of *Pseudomonas aeruginosa* have been extensively studied. This species is frequently isolated from hydrocarbon-contaminated environments and is recognized as one of the most important biological agents in the bioremediation of such sites.^63^ Increasing attention has been given to improving hydrocarbon removal by using immobilized systems containing bacteria from the *Pseudomonas* genus. For instance, Mostafa et al.^64^ evaluated the biodegradation of liquid petroleum residues by *P. aeruginosa* ATCC 9027 at various concentrations (1.0, 1.5, 2.0, and 2.5% v/v). The highest degradation efficiency was observed at 1.5% (v/v), where the strain achieved 99% degradation of polyaromatic and aliphatic hydrocarbons within 8 days of treatment. Similarly, Al-Dhabaan^65^ assessed the biodegradation of crude oil by *P. aeruginosa* isolated from oil-contaminated soil in Saudi Arabia. When cultured in liquid medium with 1% (v/v) crude oil over 14 days, the strain achieved a TPH biodegradation rate of 20%.

The strong biodegradation capacity of *P. aeruginosa* can be attributed to its well-known metabolic versatility. For example, *P. aeruginosa* ATCC 9027 has evolved enzymatic pathways that activate hydrocarbons, producing intermediates that enter central metabolic routes. Furthermore, strains such as *P. aeruginosa* PAO1 and RR1 possess the hydrolases AlkB1 and AlkB2, which are active during the stationary and exponential growth phases, respectively. These enzymes are encoded by the *alkMb* gene, whose expression is induced by exposure to *n*-alkanes with carbon chain lengths ranging from C_16_ to C_22_.^12^

In a study on the immobilization of *Pseudomonas putida* at 30 °C over 74 days, an increase in colony-forming units (CFU) within the immobilization beads was observed—from 1.1 to 1.3 CFU·g^– 1^. This increase was attributed to enhanced metabolic activity and the restricted diffusion of growth-inhibiting compounds.^66^ Similarly, when *Pseudomonas aeruginosa* strain ASW-2 was immobilized in beads and exposed to 1% (v/v) crude oil, in combination with *Exiguobacterium* sp. ASW-1, *Alcaligenes* spp. ASW-3 and ASS-1, and *Bacillus* sp. ASS-2, a biodegradation efficiency of 75% was achieved by the seventh day of treatment.^67^

The effectiveness of TPH removal by both studied strains is also linked to their capacity to produce biosurfactants. Some bacteria naturally synthesize biosurfactants that fulfill multiple physiological roles, including enhancing motility and chemotaxis, facilitating hydrocarbon localization and degradation, modifying cell membrane hydrophobicity, promoting biofilm formation, and mediating *Quorum Sensing*. Through mechanisms such as pseudosolubilization and emulsification, biosurfactants improve the bioavailability of hydrocarbons to bacteria, thereby enhancing their biodegradation.^68^

In *Pseudomonas stutzeri* AG 22, high levels of biosurfactant production—primarily rhamnolipids—have been reported, which are considered among the most effective compounds for enhancing diesel biodegradation in this species.^69^ In the case of *Chromobacterium violaceum*, the UCP 1489 strain isolated from Brazil’s Paca River has been shown to produce biosurfactants capable of reducing water surface tension from 71 mN·m^–1^ to 26 mN·m^–1^.^70^

Regarding the growth mode of *C. violaceum* ATCC 12472 in the liquid medium in the diesel sample, it was presented both planktonically and with the formation of biofilms adhered to the diesel droplets. As a result of the production of reported surfactants, it is possible to explain the growth mode of *C. violaceum* ATCC 12472 in the liquid medium in the diesel sample. Additionally, as part of the investigations carried out in the genome of *C. violaceum*, the regulation of the *Quorum Sensing* mechanism to the hmsHNFR operon has been identified for the regulation of stress conditions and the formation of biofilms.^71^

From the metabolic mechanisms of *C. violaceum* ATCC 12472, the results obtained contribute to the knowledge about the degradation capacity of hydrocarbons and contribute to the results of TPHs removal in diesel samples reported by Bassey et al.,^51^ where it is described that *C. violaceum* used hydrocarbon media as a carbon source and obtained the highest percentage of hydrocarbon degradation during 5 days of incubation in samples of gasoline, kerosene and diesel samples. However, unlike that study, in the present research work the quantification was achieved directly in the removal of TPHs and not only indirectly with the growth curve.

### 3.2 Biodegradation of Phenols by *C. violaceum* ATCC 12472

*C. violaceum* ATCC 12472 and *P. aeruginosa* ATCC 9027, both in free and immobilized forms, effectively degraded phenolic compounds over 4 days of incubation, as shown in Figure 2. Phenol concentrations significantly decreased in all biotic treatments, with reductions clearly distinguished from the abiotic controls (*p* < 0.05). The treatment with free cells of *C. violaceum* ATCC 12472 showed a somewhat lower performance. The biodegradation efficiency for this treatment lags behind the others (Figure 3A) and its biodegradation rate peaks later and lower than that of the remaining treatments (Figure 3B).

**Figure 2:**
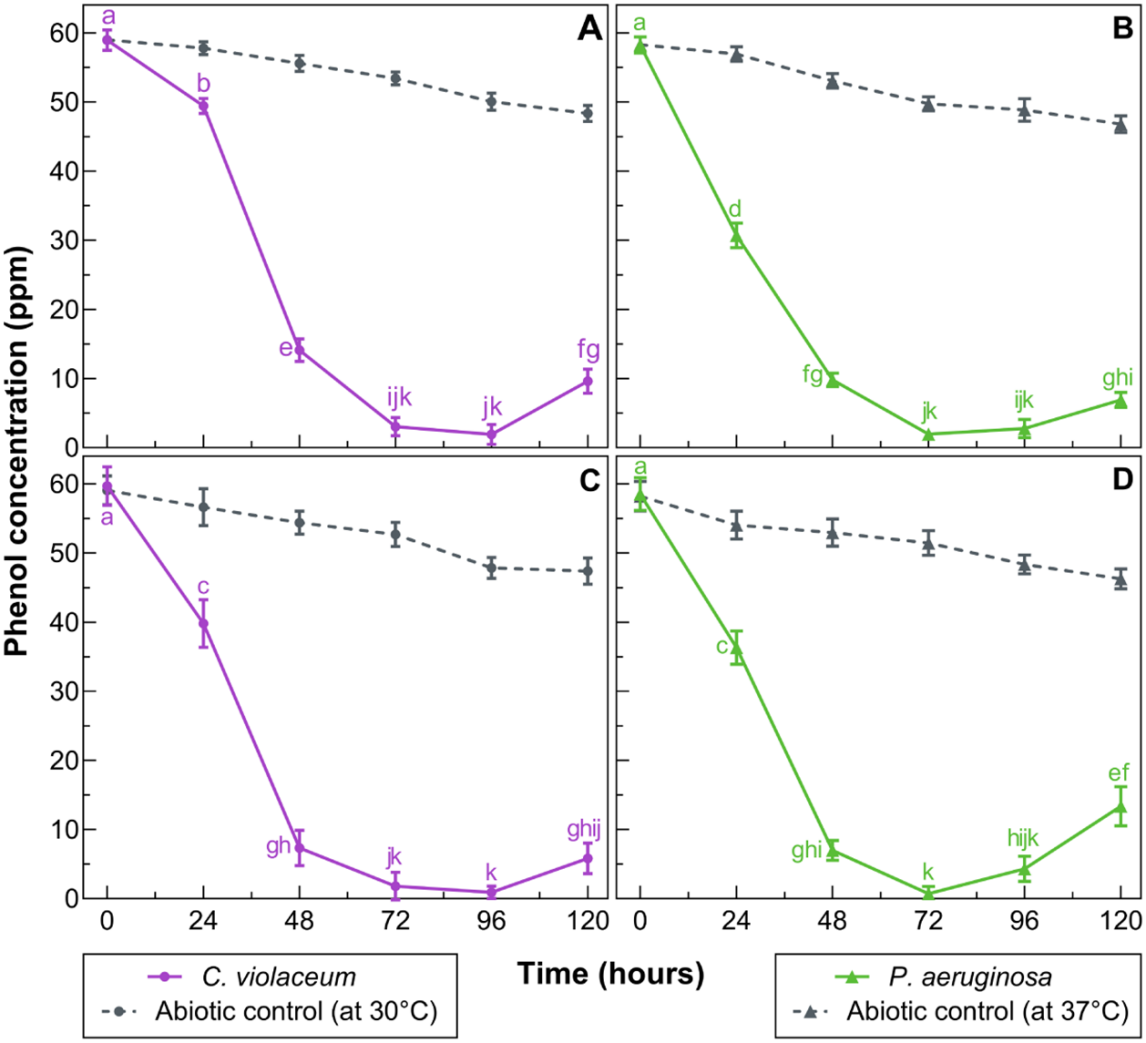
Degradation of phenols by free (A, B) and immobilized (C, D) *C. violaceum* ATCC 12472 (left) and *P. aeruginosa* ATCC 9027 (right). Error bars represent standard deviations, and letters indicate significant differences (*p* < 0.05) between time points for each strain. Points with different letters are statistically different, while shared letters indicate no significant difference.

**Figure 3:**
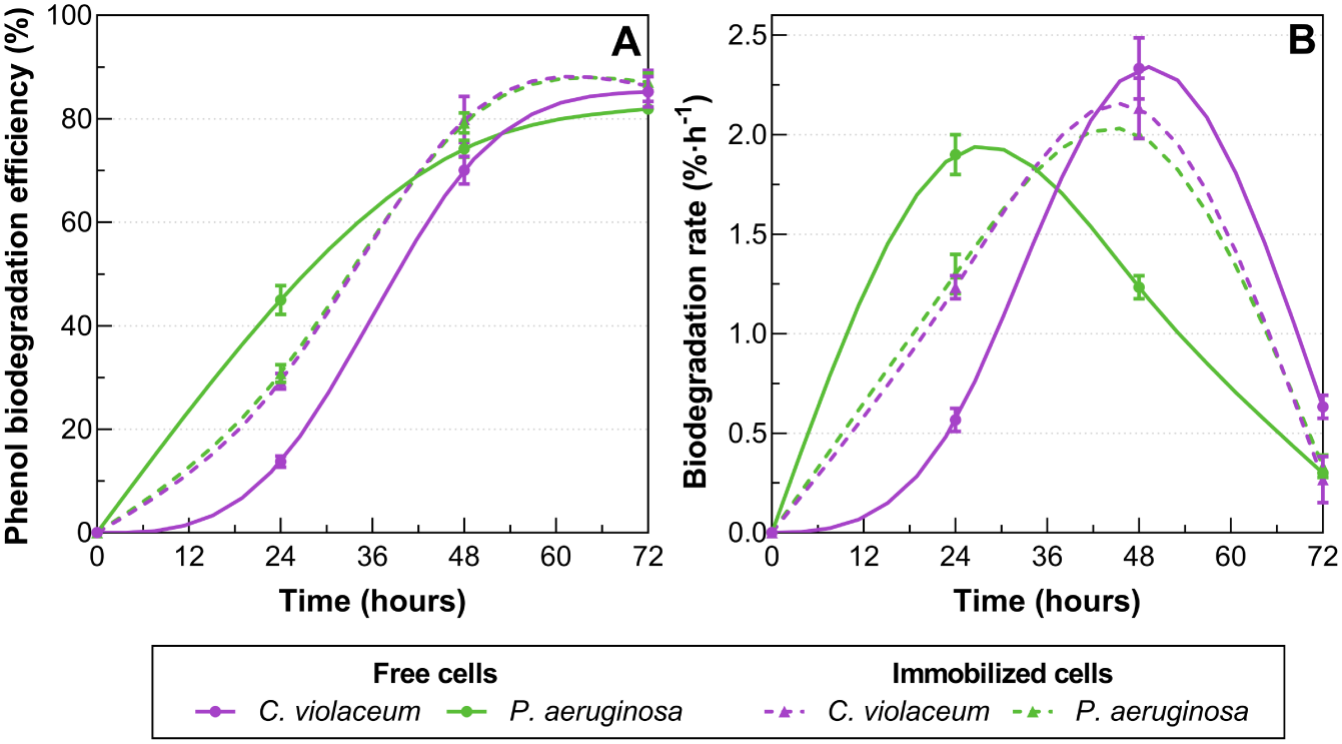
(A) Phenol biodegradation efficiency and (B) phenol biodegradation rate by free and immobilized *C. violaceum* ATCC 12472 and *P. aeruginosa* ATCC 9027. Error bars represent standard deviations.

While most treatments reached maximum phenol removal within 72 h, free *C. violaceum* required up to 96 h, indicating a slower adaptation or metabolic activation. This trend aligns with reports by Mostafa et al.,^64^ who described a lag phase for *P. aeruginosa* in petroleum-containing media during the first 24–36 h, characterized by limited substrate use and metabolite secretion. Similar growth patterns may be inferred for *C. violaceum*, explaining its delayed response.

Table 3 summarizes the phenol biodegradation percentages, where no statistically significant differences were observed between treatments with free and immobilized cells, nor between the evaluated strains. In contrast to the findings of Mohanty and Jena^72^ who reported higher biodegradation efficiencies for immobilized cells of *Pseudomonas* *sp.* NBM11 in calcium alginate beads compared to free cells, the results of this study suggest that cell immobilization of *C. violaceum* ATCC 12472 and *P. aeruginosa* ATCC 9027 did not significantly enhance phenol biodegradation capacity under the tested conditions.

**Table 3:** Percentages of Biodegradation of Phenols by Free and Immobilized Cells of *C. violaceum* ATCC 12472 and *P. aeruginosa* ATCC 9027 (Positive Control) 3 Days

Treatment	Bacterium	Biotic degradation (%)	Abiotic degradation (%)	Biodegradation (%)
Free	*C. violaceum* ATCC 12472	95 ± 5	12 ± 1	83 ± 3
	*P. aeruginosa* ATCC 9027	96 ± 3	13 ± 1	83 ± 3
Immobilized	*C. violaceum* ATCC 12472	98 ± 2	12 ± 1	86 ± 1
	*P. aeruginosa* ATCC 9027	98 ± 1	14 ± 1	85 ± 2

Figure 3A further supports this, showing no significant differences in efficiency between immobilized and free treatments, indicating that both microorganisms maintain their functional capacity regardless of immobilization. Likewise, Figure 3B shows that the biodegradation rate peaked at 24 h for free *C. violaceum*, and at 48 h for both immobilized *C. violaceum* and free and immobilized *P. aeruginosa*, confirming that the most intense metabolic activity occurred during the exponential growth phase. However, after 48 h, the degradation rate sharply declined, likely due to phenol depletion, oxygen limitation, or acidification of the medium, as suggested by Choudhury.^73^

Phenolic compounds at concentrations exceeding 80 ppm are known to be toxic and can inhibit microbial growth.^74^ At higher concentrations (250–1250 mg/L), exposure can cause significant cell death within 48 h, as observed in *P. aeruginosa* NBM11 and *P. putida* Ek II strains.^72,75^ Considering that the phenol concentrations used in this study were moderate, this may explain the absence of statistically significant differences.

Banerjee and Ghoshal^76^ reports that factors such as medium pH and alginate concentration in the beads strongly influence phenol degradation capacity. In their study, immobilized cells of *Bacillus cereus* AKG1MTCC9817 and AKG2MTCC9818 showed the highest degradation rates at pH 6.7 and 6.9, respectively, which was attributed to the polyanionic nature of the alginate matrix, capable of reducing the effective H^+^ concentration around immobilized cells. Moreover, it has been reported that higher alginate concentrations significantly decrease the diffusion coefficient of phenol, exerting a limiting effect on its biodegradation rate.

According to the study by Perpetuo et al.,^77^ *C. violaceum* CVT8, as well as recombinant *Escherichia coli* expressing the Cvmp gene, achieved complete phenol removal at a concentration of 0.4 ppm by the fifth day of incubation. By the fourth day, a removal efficiency of 63% was observed in cultures with free cells. The Cvmp gene encodes a phenol monooxygenase, an enzyme responsible for initiating the degradation pathway that enables *C. violaceum* to use phenol as a carbon source. This metabolic process begins with the hydroxylation of the aromatic ring at the ortho or para position, producing catechol or hydroquinone, respectively. These intermediates are then subjected to meta-cleavage by C2,3O, a key enzyme in the aerobic degradation of phenolic compounds.

C2,3O belongs to the family of intradiol and extradiol dioxygenases and plays a fundamental role in the ring-cleavage step of the biodegradation pathway. This enzyme has been widely identified in several Gram-negative genera, including *Pseudomonas*, *Sphingomonas*, *Acinetobacter*, *Ralstonia*, *Burkholderia*, and *Stenotrophomonas*, as well as in Gram-positive bacteria such as *Nocardia*, *Rhodococcus*, and *Bacillus*.^36^ The presence and activity of this enzyme are essential for the effective breakdown of aromatic rings and are closely associated with the biodegradation capabilities observed in this study.

Figure 4 shows the enzymatic activity of C2,3O by free cells over 120 h. *P. aeruginosa* and *C. violaceum* reached maximum activity between 24 h (∼1.8 U/mL) and 48 h (∼2.0 U/mL). In both strains, the highest enzyme activity coincides with the exponential growth phase, supporting the role of C2,3O in the initial degradation of phenol via the meta-cleavage pathway. Based on this result, the present study suggests that the first peak observed at 24 h may represent the optimal time point for scale-up procedures under the evaluated conditions.

**Figure 4:**
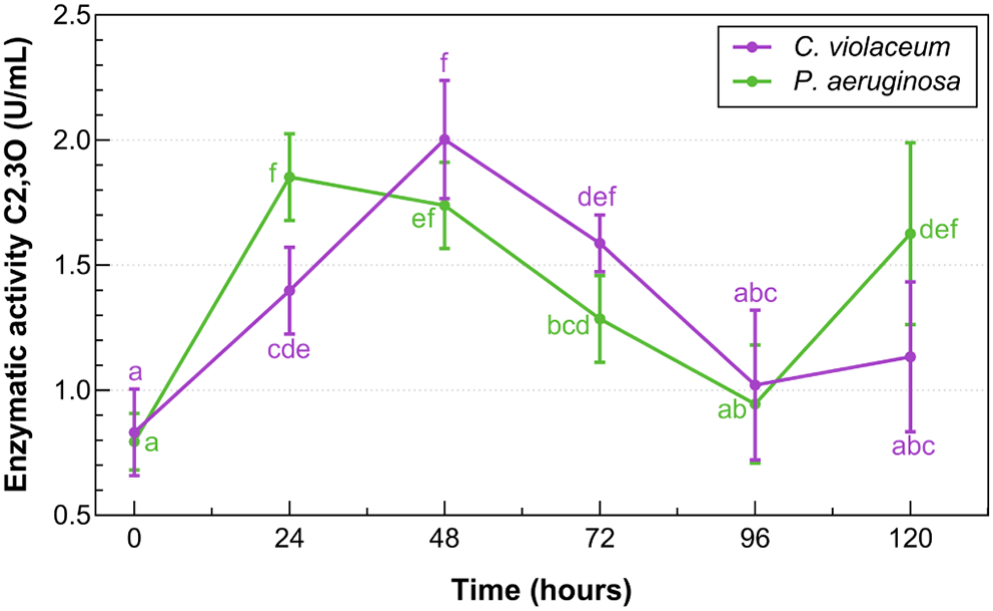
Enzymatic activity of C2,3O by free *C. violaceum* ATCC 12472 and *P. aeruginosa* ATCC 9027. Error bars represent standard deviations, and letters indicate significant differences (*p* < 0.05) between time points for each strain. Points with different letters are statistically different, while shared letters indicate no significant difference.

In the case of *Pseudomonas*, based on what was reported by Wei et al.,^78^ enzyme activity tests in *Pseudomonas* sp., showed that most isolates growing with Polycyclic Aromatic Hydrocarbons as the only source of carbon and energy had an active C2,3O. More recently, Mohanty and Jena^72^ identified that from 24 to 48 h there was the greatest increase in biomass, similar to the growth obtained for the strain of *P. aeruginosa* ATCC 9027 in the present study.

In the case of *C. violaceum*, a study on the CVT8 strain suggests that the primary product of phenol degradation is hydroquinone.^75^ The C2,3O enzyme, which plays a central role in the upper metabolic pathway by cleaving the carbon–carbon bond at the 2,3 (meta) position of catechol, is essential for phenol degradation during bacterial growth. This explains why the highest C2,3O activity was observed during the exponential growth phase in both microorganisms evaluated in this study.

Throughout the biodegradation process, the decrease in oxygen concentration and pH has been reported in studies of phenol degradation, metabolized by bacteria in a mixture of *Pseudomonadaceae*, *Vibrionaceae*.^73^ On the other hand, the enzyme C2,3O has shown a negative effect when the solution varies to alkaline, where its activity is decreased,^78^ and after 48 h in both cultures, when there is a lower concentration of oxygen and variation in pH, the activity of the enzyme is reduced in the following days.

## 4 Conclusion

This study demonstrated that *Chromobacterium violaceum* ATCC 12472 is capable of efficiently degrading diesel hydrocarbons and phenolic compounds, showing a degradation performance comparable to *Pseudomonas aeruginosa* ATCC 9027, a well-known model organism for hydrocarbon bioremediation. Given its robust metabolic activity, stress tolerance, and adaptability to hydrocarbon-rich environments, *C. violaceum* ATCC 12472 emerges as a promising candidate for the treatment of diesel-contaminated process waters.

The immobilization of bacterial cells in calcium alginate beads significantly enhanced the removal efficiency of total petroleum hydrocarbons for both strains, although this effect was not observed for phenols. For total petroleum hydrocarbons, this improvement can be attributed to the increased biomass density, protection from toxic compounds, and sustained metabolic activity provided by the immobilization matrix. These findings confirm that cell immobilization is a viable strategy to improve hydrocarbon biodegradation rates in practical applications. In contrast, regarding phenols, this study highlights the need to evaluate factors such as medium pH and alginate bead concentration in order to determine whether cell immobilization could enhance the biodegradation efficiency of these compounds.

The results also highlight the usefulness of controlled laboratory bioassays to evaluate microbial strains for their bioremediation potential. *C. violaceum* ATCC 12472 demonstrated key traits relevant to environmental applications, including the ability to form biofilms, produce biosurfactants, tolerate high hydrocarbon concentrations, and activate catabolic pathways such as those involving catechol 2,3-dioxygenase.

## Supplementary Material



## Notes

Calibration curves for bacterial suspensions and phenol quantification, along with detailed statistical analyses supporting the study’s results. It includes ANOVA and post hoc tests for the growth kinetics of *Chromobacterium violaceum* in diesel and LB broth, comparisons of kinetic parameters, phenol degradation by free and immobilized cells of *C. violaceum* and *P. aeruginosa*, and C2,3O enzyme activity; complete statistical and methodological support for the findings presented in the main article (PDF)
