# Supplementary material for: Biodegradation of Diesel by Free and Immobilized Chromobacterium violaceum
Source: ACS Omega. 2025 Nov 12;10(46):56255–64. doi: 10.1021/acsomega.5c07846 (PMC12658640; doi:10.1021/acsomega.5c07846)
Supplement: Supplementary file 1 [file ao5c07846_si_001.pdf]

SUPPLEMENTARY MATERIALS.

Biodegradation of Diesel by Free and Immobilized *Chromobacterium violaceum*

Jhonson Sebastian Arenas Soler (sebastian21arenas@gmail.com) [a], Nathaly Rivera (nathaly1812@gmail.com) [a], Francy Janeth Méndez Casallas (fmendez@unisalle.edu.co) [a,b], Boris Galvis (boris.galvis@correounivalle.edu.co) [c, *].

[a] Programa de Ingeniería Ambiental y Sanitaria. Universidad de La Salle, (https://www.lasalle.edu.co/), Bogotá, 111711, Colombia.

[b] Facultad de Ciencias de la Salud. Universidad Colegio Mayor de Cundinamarca, (https://www.universidadmayor.edu.co/) Bogotá, 110911, Colombia

[c] Sanitary and Environmental Engineering Program. Universidad del Valle, (https://eidenar.univalle.edu.co/) Cali, 760042, Colombia.

[*] Email: boris.galvis@correounivalle.edu.co

CALIBRATION CURVES

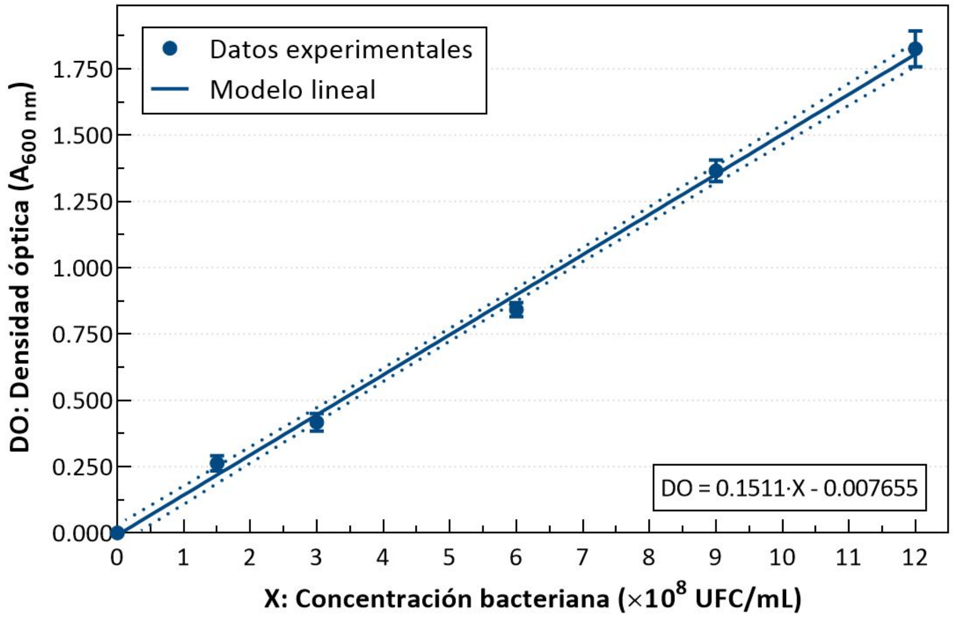

Figure S1. Standardization curve of bacterial suspensions evaluated with the linear regression model (R2 = 0.9913). **Dotted lines correspond to the model's 95% confidence interval.*

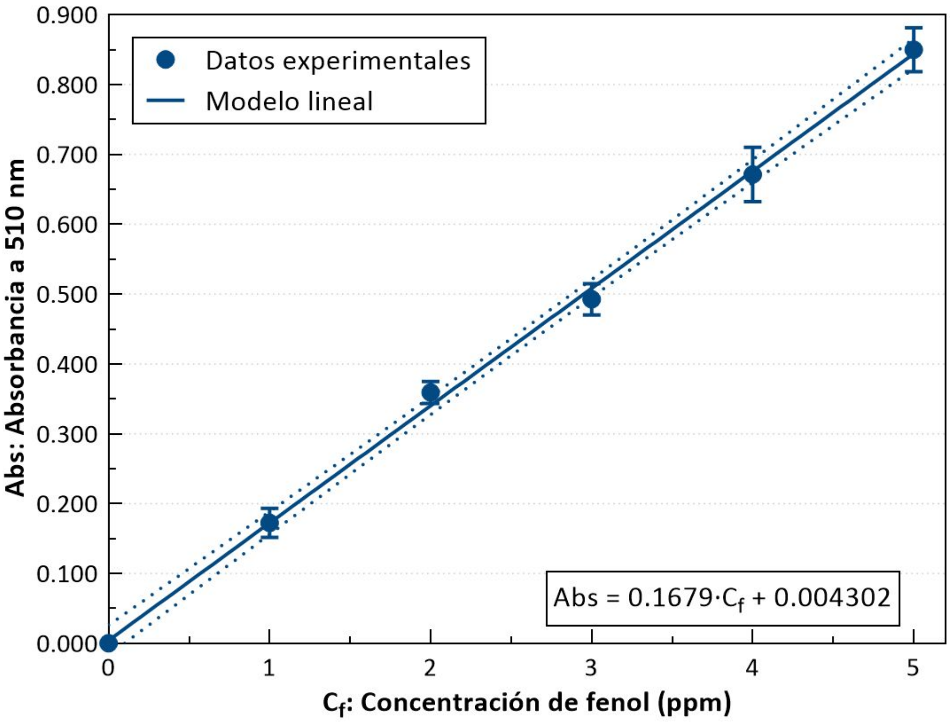

Figure S2. Phenol calibration curve evaluated with the linear regression model (R2 = 0.9936). **Dotted lines correspond to the model's 95% confidence interval.*

STATISTICAL ANALYSIS

Growth kinetics of *C. violaceum* ATCC 12472 at different diesel concentrations

Table S1: Two-factor ANOVA (time and treatment) in the growth kinetics of *C. violaceum* ATCC 12472 in diesel.

Inter-subject effects tests

Dependent variable: Densidad_óptica

Origin	Type III of Sum of Squares	Gl	Quadratic mean	F	Gis.	Partial eta squared
Corrected model	0.940A	83	0.011	398.899	0.000	0.994
Intersection	1.795	1	1.795	63214.485	0.000	0.997
Time	0.440	13	0.034	1192.855	0.000	0.988
Treatment	0.433	5	0.087	3047.313	0.000	0.987
Time * Treatment	0.137	65	0.002	74.256	0.000	0.961
Error	0.006	196	2.839E-5			
Total	2.478	280				
Total corrected	0.945	279				

[a.] *R squared = 0.994 (R squared adjusted = 0.992)*

Table S2: Post hoc test for the different treatments evaluated in the growth kinetics of *C. violaceum* ATCC 12472 in diesel.

Optical Density

Treatment	N	Subset		
		c	b	to
Abiotic control	70	0.00601		
7.5 %	42		0.09302	
5 %	42		0.09383	
2.5 %	42			0.09829
1 %	42			0.09874
10 %	42			0.09926
Gis.		1.000	0.472	0.417

The means for the groups in the homogeneous subsets are displayed.
It is based on the observed averages.
The error term is the mean square (Error) = 2.839E-5.
[a] *Use the sample size of the harmonic mean = 45,000.*
[b] *Group sizes are not the same. The harmonic mean of the group sizes is used. Type I error levels are not guaranteed.*
[c] *Alpha = 0.05.*

Table S3: Post hoc test for the Time factor*Treatment of the growth kinetics of *C. violaceum* ATCC 12472 in diesel.

Optical Density

Time* Treatment	N	Subset for alpha = 0.05										
		to	b	c	d	and	f	g	h	i	j	k
Day0*2.5%	3	0.009										
Day0*7.5%	3	0.009										
Day0*1%	3	0.010										
Day1*7.5%	3	0.010										
Day1*2.5%	3	0.011										

Time* Treatment	N	Subset for alpha = 0.05										
		to	b	c	d	and	f	g	h	i	j	k
Day0*10%	3	0.011										
Day0*5%	3	0.012										
Day1*1%	3	0.012										
Day1*5%	3	0.014										
Day1*10%	3	0.014										
Day2*5%	3	0.015										
Day2*10%	3	0.015										
Day2*1%	3	0.016										
Day2*7.5%	3	0.016										
Day2*2.5%	3	0.020										

Day3*10%	3		0.071									
Day3*1%	3		0.078									
Day3*7.5%	3		0.080									
Day3*5%	3			0.090								
Day4*7.5%	3				0.102							
Day4*10%	3				0.102							
Day4*5%	3				0.109							
Day10*2.5%	3				0.112							
Day5*5%	3					0.123						
Day5*7.5%	3					0.123						
Day4*1%	3					0.125						
Day5*10%	3					0.126						
Day3*2.5%	3					0.128						
Day10*7.5%	3					0.128						
Day10*5%	3					0.129						
Day10*1%	3					0.130						
Day9*2.5%	3						0.139					
Day4*2.5%	3						0.140					
Day7*5%	3						0.141	0.141				
Day10*10%	3						0.141	0.141				
Day5*1%	3						0.142	0.142				
Day9*5%	3						0.143	0.143	0.143			
Day9*7.5%	3						0.144	0.144	0.144	0.144		
Day8*5%	3						0.147	0.147	0.147	0.147	0.147	
Day8*7.5%	3						0.147	0.147	0.147	0.147	0.147	
Day5*2.5%	3						0.147	0.147	0.147	0.147	0.147	
Day7*7.5%	3						0.149	0.149	0.149	0.149	0.149	0.149
Day9*1%	3						0.149	0.149	0.149	0.149	0.149	0.149
Day8*2.5%	3						0.151	0.151	0.151	0.151	0.151	0.151
Day7*1%	3							0.153	0.153	0.153	0.153	0.153
Day7*10%	3								0.154	0.154	0.154	0.154
Day7*2.5%	3									0.155	0.155	0.155
Day8*1%	3										0.157	0.157
Day8*10%	3										0.158	0.158
Day9*10%	3											0.160
Gis.		0.065	0.070	1.000	0.070	0.225	0.055	0.053	0.050	0.066	0.068	0.055

The means for the groups in the homogeneous subsets are displayed.

[a.] Use the sample size of the harmonic mean = 3,000.

Growth kinetics of C. violaceum ATCC 12472 in LB broth

Table S4: ANOVA of the optical density in time of the growth kinetics of *C. violaceum* ATCC 12472 in broth LB.

ANOVA<br>optical density					
	Sum of squares	Gl	Quadratic mean	F	Gis.
Between groups	2.526	24	0.105	139.557	0.000
Within groups	0.038	50	0.001		
Total	2.564	74			

Table S5: Post hoc test for the optical density over time of the growth kinetics of *C. violaceum* ATCC 12472 in broth LB.

optical density									
Time	N	Subset for alpha = 0.05							
		to	B	c	d	and	f	g	h
0	3	0.03644							
1	3	0.05447							
2	3	0.07416	0.07416						
3	3		0.11582						
4	3			0.29835					
5	3				0.35879				
6	3					0.45156			
7	3					0.48087	0.48087		
8	3						0.50309	0.50309	
9	3							0.54011	0.54011
10	3							0.54978	0.54978
11	3								0.55980
12	3								0.56277
22	3								0.57078
15	3								0.57159
13	3								0.57199
16	3								0.57204
18	3								0.57284
24	3								0.57365
14	3								0.57577
20	3								0.57577
23	3								0.57577
19	3								0.57662
17	3								0.57788
21	3								0.57828
Gis.		0.118	0.069	1.000	1.000	0.197	0.327	0.053	0.168

The means for the groups in the homogeneous subsets are displayed.

[a.] *Use the sample size of the harmonic mean = 3,000.*

Kinetic growth parameters of C. violaceum ATCC 12472 in diesel and LB broth

Table S6: ANOVA of the kinetic growth parameters of *C. violaceum* ATCC 12472 in diesel and LB broth.

		Sum of squares	Gl	Quadratic mean	F	Gis.
Vmax	Between groups	1.291	5	0.258	149.036	0.000
	Within groups	0.021	12	0.002		
	Total	1.311	17			
Tg	Between groups	373.046	5	74.609	18.491	0.000
	Within groups	48.418	12	4.035		
	Total	421.464	17			
M	Between groups	0.373	5	0.075	2494.351	0.000
	Within groups	0.000	12	0.000		
	Total	0.373	17			

Table S7: Post hoc test for the kinetic growth parameters of *C. violaceum* ATCC 12472 in diesel and LB broth.

Max Vel

Treatment	N	Subset for alpha = 0.05		
		to	B	C
10 %	3	0.0490		
7.5 %	3	0.0575		
1 %	3	0.0889	0.0889	
5 %	3	0.0968	0.0968	
2.5 %	3		0.1546	
LB broth	3			0.8020
Gis.		0.216	0.090	1.000

Tg

Treatment	N	Subset for alpha = 0.05		
		c	b	to
LB broth	3	0.8716		
2.5 %	3		4.5480	
5 %	3		7.7732	
1 %	3		7.9656	
7.5 %	3			12.5942
10 %	3			14.3609
Gis.		1.000	0.070	0.303

M

Treatment	N	Subset for alpha = 0.05			
		To	b	c	d
5 %	3	0.12363			
7.5 %	3		0.13527		
1 %	3		0.13872	0.13872	
2.5 %	3		0.14305	0.14305	
10 %	3			0.14796	
LB broth	3				0.52327
Gis.		1.000	0.123	0.071	1.000

The means for the groups in the homogeneous subsets are displayed.
[a] *Use the sample size of the harmonic mean = 3,000.*

Degradation of phenols by free and immobilized cells of C. violaceum ATCC 12472 and P. aeruginosa ATCC 9027

Table S8: Two-factor ANOVA (time and treatment) in the degradation of phenols by free and immobilized cells of *C. violaceum* ATCC 12472 and *P. aeruginosa* ATCC 9027.

Inter-subject effects tests

Dependent variable: Phenols

Origin	Type III of Sum of Squares	Gl	Quadratic mean	F	Gis.
Corrected model	63094.710a	39	1617.813	232.306	0.000
Intersection	172946.992	1	172946.992	24833.904	0.000
Day	20998.813	4	5249.703	753.818	0.000
Treatment	30044.388	7	4292.055	616.307	0.000
Day * Treatment	12051.510	28	430.411	61.804	0.000
Error	557.132	80	6.964		
Total	236598.834	120			
Total corrected	63651.842	119			

a. R squared = 0.991 (R squared adjusted = 0.987)

Table S9: Post hoc test for treatments evaluated in the degradation of phenols by free and immobilized cells of *C. violaceum* ATCC 12472 and *P. aeruginosa* ATCC 9027.

Phenols

Treatment	N	Subset		
		1	2	3
Free P.aeruginosa	15	20.580		
Immobilized P.aeruginosa	15	20.691		
Immobilized C.violceum	15	21.763		
Free C.violaceum	15		25.858	
Immobilized Control 37 °C	15			52.530
Free Control 37 °C	5			53.494
Immobilized Control 30 °C	15			54.328
Free Control 30 °C	15			54.464
Gis.		0.252	1.000	0.069

The means for the groups in the homogeneous subsets are displayed.
It is based on the observed averages.
The error term is the mean square (Error) = 6.964.
to. Use the sample size of the harmonic mean = 15,000.
b. Alpha = 0.05.

Table S10: Post hoc test for the Time factor*Treatment of phenol degradation by free and immobilized cells of *C. violaceum* ATCC 12472 and *P. aeruginosa* ATCC 9027.

Phenols

Treament time	N	Subset for alpha = 0.05							
		h	g	f	and	d	c	b	to
Day 4 immobilized C.violaceum	3	0.699							
Day 4 immobilized P.aeruginosa	3	0.759							

Day 3 immobilized P.aeruginosa	3	0.909							
Day 3 immobilized C.violaceum	3	1.290							
Day 4 Free P.aeruginosa	3	1.590							
Day 4 Free C.violaceum	3	1.910	1.910						
Day 3 Free P.aeruginosa	3	2.524	2.524	2.524					

Tiempo_Tratamiento	N	Subset for alpha = 0.05							
		h	g	f	and	d	c	b	to
Day 3 Free C.violaceum	3	3.010	3.010	3.010					
Day 2 Immobilized P.aeruginosa	3		6.952	6.952					
Day 2 Immobilized C.violaceum assets	3			7.332					
Day 2 P.aeruginosa_Libre	3			7.813					
Day 2 Free C.violaceum	3				16.012				
Day 1 Free P.aeruginosa	3					32.710			
Day 1 Immobilized P.aeruginosa	3					36.313	36.313		
Day Immobilized C.violaceum	3						39.794		
Day1 Free C.violaceum	3							49.418	
Day 0 Free P.aeruginosa	3								58.261
Day 0 Immobilized P.aeruginosa	3								58.521
Day 0 Free C.violaceum	3								58.942
Day 0 Immobilized C.violaceum	3								59.702
Gis.		0.411	0.057	0.051	1.000	0.137	0.150	1.000	0.586

The means for the groups in the homogeneous subsets are displayed.
to. Use the sample size of the harmonic mean = 3,000.

C2,3O enzyme activity by free cells of C. violaceum ATCC 12472 and P. aeruginosa ATCC 9027

Table S11: ANOVA of C2,3O enzyme activity by cell-free *C. violaceum* ATCC 12472 and *P. aeruginosa* ATCC 9027.

ANOVA

Actividad_enzimática

	Sum of squares	Gl	Quadratic mean	F	Gis.
Between groups	5.235	9	0.582	15.324	0.000
Within groups	0.759	20	0.038		
Total	5.995	29			

Table S12: Post hoc test for C2,3O enzyme activity by cell-free *C. violaceum* ATCC 12472 and *P. aeruginosa* ATCC 9027.

Enzymatic Activity

Treatment Day	N	Subset for alpha = 0.05

		To	b	c	d	and	f
Day 0 P.aeruginosa	3	0.7933					
Day 0 C.violaceum	3	0.8300					
Day 4 P.aeruginosa	3	0.9447	0.9447				
Day 4 C.violaceum	3	1.0200	1.0200				
Day 3 P.aeruginosa	3		1.2833	1.2833			
Day 1 C.violaceum	3			1.4000	1.4000		
Day 3 C.violaceum	3			1.5867	1.5867	1.5867	
Day 2 P.aeruginosa	3				1.7400	1.7400	1.7400
Day 1 P.aeruginosa	3					1.8500	1.8500
Day 2 C.violaceum	3						2.0033
Gis.		0.206	0.056	0.085	0.055	0.132	0.132

The means for the groups in the homogeneous subsets are displayed.
to. Use the sample size of the harmonic mean = 3,000.
